# Phenotypic consequences of somatic mutations in the ataxia-telangiectasia mutated gene in non-small cell lung cancer

**DOI:** 10.18632/oncotarget.11845

**Published:** 2016-09-02

**Authors:** Anika Maria Weber, Neele Drobnitzky, Aoife Maire Devery, Sivan Mili Bokobza, Richard A. Adams, Timothy S. Maughan, Anderson Joseph Ryan

**Affiliations:** ^1^ Department of Oncology, Cancer Research UK and Medical Research Council Oxford Institute for Radiation Oncology, University of Oxford, Oxford, UK; ^2^ Institute of Cancer & Genetics, Cardiff University, School of Medicine, Cardiff, UK

**Keywords:** non-small cell lung cancer (NSCLC), ataxia-telangiectasia mutated (ATM), missense mutation, DNA damage response (DDR), radiosensitizer

## Abstract

Mutations in the Ataxia-telangiectasia mutated (ATM) gene are frequently found in human cancers, including non-small cell lung cancer (NSCLC). Loss of ATM function confers sensitivity to ionising radiation (IR) and topoisomerase inhibitors and may thus define a subset of cancer patients that could get increased benefit from these therapies. In this study, we evaluated the phenotypic consequences of ATM missense changes reported in seven NSCLC cell lines with regard to radiosensitivity and functionality of ATM signalling. Our data demonstrate that only 2/7 NSCLC cell lines (H1395 and H23) harbouring ATM missense mutations show a functional impairment of ATM signalling following IR-exposure. In these two cell lines, the missense mutations caused a significant reduction in ATM protein levels, impairment of ATM signalling and marked radiosensitivity. Of note, only cell lines with homozygous mutations in the ATM gene showed significant impairment of ATM function. Based on these observations, we developed an immunohistochemistry-based assay to identify patients with loss or reduction of ATM protein expression in a clinical setting. In a set of 137 NSCLC and 154 colorectal cancer specimens we identified tumoral loss of ATM protein expression in 9.5% and 3.9% of cases, respectively, demonstrating the potential utility of this method.

## INTRODUCTION

Lung cancer is the most common cancer type and, at the same time, has one of the lowest survival outcomes, rendering it the leading cause of cancer-related mortality worldwide [1]. Most lung cancer patients are treated with DNA-damaging modalities such as chemotherapy and ionising radiation (IR). Although effective, these treatments generally lack selectivity towards cancer cells resulting in normal tissue toxicity and potentially severe side effects, which are limiting factors for both the dose and duration of therapy [2, 3]. These limitations are one of the reasons why these genotoxic therapies are rarely curative and a substantial amount of research in recent years has focused on the identification of potential combination approaches and new molecular targets that may enhance the anti-tumour efficacy of chemo- or radiotherapy without increasing normal tissue toxicity. A promising target for this approach is the kinase Ataxia-telangiectasia mutated (ATM).

ATM is one of the principal mediators of the DNA damage response (DDR), a complex network of signalling pathways that mediates the detection and repair of DNA damage and the enforcement of DNA damage-induced cell cycle checkpoints [4–6]. Germline mutations in the ATM gene are responsible for the autosomal recessive human hereditary disorder Ataxia-telangiectasia (A-T). This disease, caused by a loss of ATM function, is characterised by progressive cerebellar degeneration, telangiectasia, immunodeficiency, growth retardation, genomic instability, cancer susceptibility and profound sensitivity to IR [4, 7–9]. Notably, somatic mutations in the ATM gene have been reported in a broad range of human cancers including colorectal [10], breast [11] and haematopoietic cancers [12, 13] as well as in 5.1-9.1% of lung cancer cases [14, 15]. Considering the substantial sensitivity to IR, radiomimetic drugs and topoisomerase inhibitors observed in cells derived from A-T patients [7, 16, 17], tumoral loss of ATM function may define a subset of patients that could get increased benefit from such treatments.

**Figure 1:**
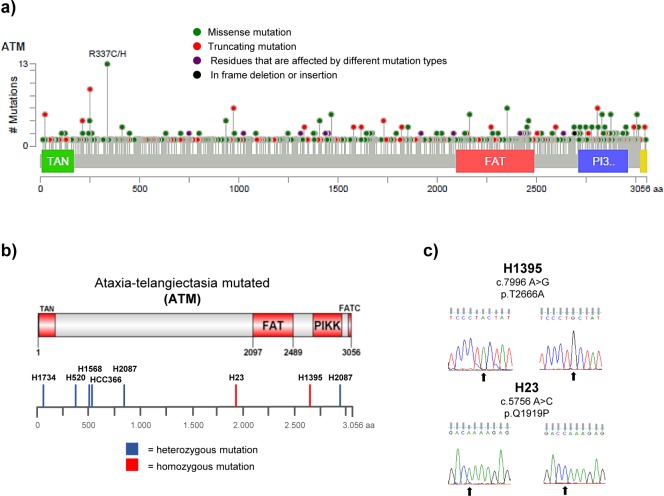
Distribution and types of ATM mutations in different cancer types FAT (FRAP-ATM-TRRAP domain), FATC (FAT C-terminal domain), PIKK (phosphatidylinositol 3-kinase-related kinase domain), TAN (Tel1/ATM N-terminal motif).**a.** Schematic representation of the ATM protein with the positions of 722 ATM mutations reported in 108 representative cancer genomics studies highlighted below. Data analysis and visualisation were performed using the cBioPortal Cancer Genomics tool (http://www.cbioportal.org; access on 22.01.2016) [19, 20] and the results shown here are in part based upon data generated by the TCGA Research Network:http://cancergenome.nih.gov/. **b.** Distribution of ATM missense changes in NSCLC cell lines. Schematic representation of the ATM protein displaying the positions of homo- or heterozygous ATM missense changes reported in the 7 NSCLC cell lines used in this study. **c.** DNA sequencing results showing the presence of a homozygous c.7996 A > G (p.T2666A) and c.5756 A > C (p.Q1919P) missense mutation (arrows) in the ATM gene in the H1395 and H23 cell line, respectively. Wildtype sequences derived from H1299 cells are shown on the left.

Amongst the cancer-associated ATM mutations are truncating, frame-shift or splice site mutations, which are likely to affect ATM function and/or protein expression (Figure 1a) [18]. However, a considerable proportion (~70%) of the ATM mutations reported in cancer to date are missense changes, which lead to the substitution of a single amino acid. These mutations arise across the entire length of the ATM protein, with no apparent hotspots (with the possible exception of the R337 site; Figure 1a) [19, 20]. Considering the large size of the ATM gene (150 kb of genomic DNA) and the characteristic genomic instability of cancer cells, it is likely that a proportion of the occurring missense mutations are neutral passenger mutations [21]. Distinguishing such passenger mutations from deleterious missense mutations is currently not possible without functional studies. An additional hindrance in the assessment of the functional consequences of ATM mutations is the evaluation of zygosity, as cancer cells are frequently aneuploid and even deleterious mutations might not result in any phenotypic consequences if one or several wildtype (wt) alleles are present.

The studies presented here were designed to address two issues. Firstly, given that most cancer-associated ATM mutations are missense variants whose functional consequences have not yet been elucidated, we performed phenotypic studies in a panel of seven NSCLC cell lines, which have been reported to harbour missense changes in the ATM gene (Table 1). We demonstrate that certain ATM missense changes lead to a loss of ATM protein expression and functional impairment of ATM. However, only 2/7 ATM-mutated cell lines (H23 and H1395) exhibited a gross functional impairment of ATM. In contrast, the ATM missense changes in the remaining five cell lines (HCC-366, H1568, H1734, H520 and H2087) appeared to have no or only limited impact on overall ATM function in response to IR. These observations can likely be attributed to differences in zygosity as we found that only in those cases where ATM missense mutations were homozygous did cell lines display phenotypic changes characteristic of a loss of ATM function.

**Table 1:** ATM missense changes reported in NSCLC cell lines and predicted functional consequences based on PolyPhen scoring

Cell line	Sequence change	Amino acid substitution(zygosity)	Location	PolyPhen-2 assessment (score)
H2087	c.2542G>C	p.E848Q(heterozygous)	N-terminal	benign (0.025)
H2087	c.8895G>C ^†^	p.L2965F(heterozygous)	just C-terminal of PI3K domain	probably damaging (0.992)
H23	c.5756A>C	p.Q1919P(homozygous)	FAT domain	probably damaging (1.000)
H1395	c.7996A>G	p.T2666A(homozygous)	just N-terminal of PI3K domain	probably damaging (1.000)
H520	c.1147C>G	p.P383A(heterozygous)	N-terminal	benign (0.000)
H1734	c.194A>C	p.Q65P(heterozygous)	N-terminal	probably damaging (0.987)
H1568	c.1516-1517GG>TT	p.G506F(heterozygous)	N-terminal	possibly damaging (0.842)
HCC-366	c.1600C>G	p.P534A(heterozygous)	N-terminal	probably damaging (1.000)

† We were unable to validate the presence of this mutation, as Sanger sequencing showed homozygosity for the wildtype sequence in this position (see Figure S1).

Secondly, we aimed to address the question, how patients with loss or impairment of ATM activity could be identified in a clinical setting. For this purpose, we analysed ATM protein expression by immunohistochemistry (IHC) in human NSCLC and colorectal cancer (CRC) specimens. We found that ATM protein expression was frequently lost or reduced in cancer cells (but not stromal cells), suggesting that IHC analysis of ATM protein expression may constitute a valuable tool to identify patients with loss of ATM function.

## RESULTS AND DISCUSSION

### ATM missense mutations found in NSCLC can lead to a reduction in ATM protein levels and impairment of the ATM signalling pathway

Advancements in DNA sequencing technologies have allowed for the identification of numerous genes and gene products crucially involved in cancer development and for many oncogenes or tumour suppressor genes, the functional consequences of recurrent hotspot mutations have been well characterised. For ATM, a putative tumour suppressor, this is not the case. Somatic mutations in the ATM gene have been identified in a range of cancer types, including NSCLC, and also in several established and widely used NSCLC cell lines [22–24]. We selected seven of those cell lines, which have been reported to harbour homo- (H23 and H1395) or heterozygous (HCC-366, H1568, H1734, H2087 and H520) ATM missense substitutions (see Figure 1b, and Table 1). We verified the presence of the reported mutations by Sanger sequencing (Figure 1c and S1) and calculated the PolyPhen-2 (Polymorphism phenotyping version 2) scores for the missense changes [25]. PolyPhen-2 is a software tool, which uses sequence- and structure-based information to predict the possible impact of an amino acid substitution on the structure and function of a human protein. With the exception of the c.8895 G > C (p.L2965F) mutation reported in H2087, the presence of all previously reported mutations could be validated. Several of the identified missense changes were predicted to affect protein function based on the PolyPhen algorithm (Table 1).

Studies on the characterisation of cellular defects caused by ATM-deficiency in cells derived from Ataxia-telangiectasia patients have led to the discovery of phenotypes and biomarkers associated with functional impairment of ATM [4]. To study the phenotypic consequences of the identified ATM missense substitutions with regard to radiosensitivity and functionality of ATM signalling following IR-exposure, we first determined total ATM protein levels, as well as ATM kinase activity, following exposure of the cells to 10 Gy IR by western blot analysis. Widely used biomarkers to evaluate activity of the ATM signalling pathway include ATM autophosphorylation at serine 1981, which is considered to be associated with ATM activation [26], as well as IR-induced phosphorylation of the ATM downstream targets KAP1 (S824), SMC1 (S966), NBS1 (S343), p53 (S15) and CHK2 (Thr68) [27, 28]. In addition to the ATM-mutated cell lines, the ATM wild-type cell lines COR-L105, A549 and H1299 were used as controls. Levels of the respective total and phospho-proteins in these cell lines served as reference levels for normal ATM kinase activity. The fibroblast cell line GM05849, which was derived from an A-T patient and exhibits no ATM protein expression, was used as ATM-negative control [29].

**Figure 2:**
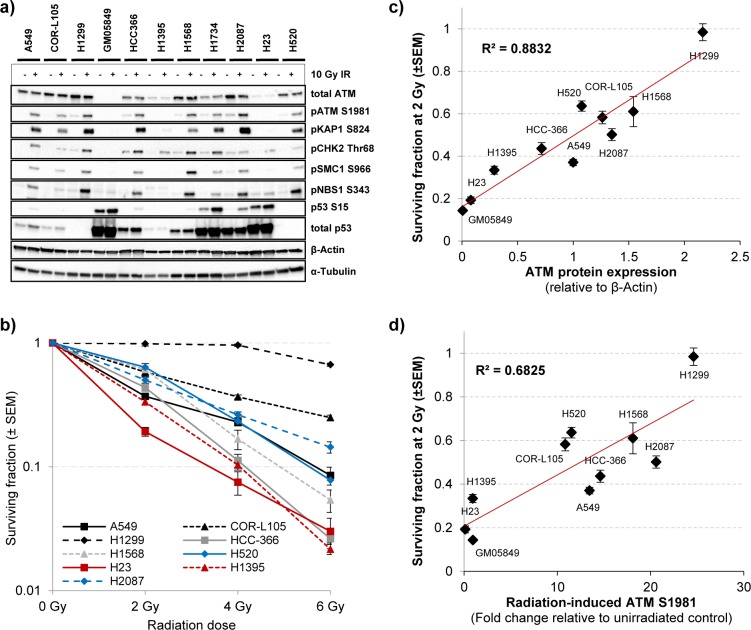
The effect of ATM missense changes on ATM protein levels, kinase activity and cellular radiosensitivity **a.** To assess the activity of the ATM signalling pathway following exposure of cells to IR, three ATM wildtype and seven ATM mutated NSCLC cell lines were mock-irradiated (control) or irradiated with 10 Gy followed by preparation of protein lysates 60 min after IR. The A-T fibroblast cell line GM05849 served as ATM-negative control. ATM autophosphorylation (S1981) and phosphorylation of several ATM downstream targets were monitored by western blot analysis. β-Actin and α-Tubulin were measured to ensure equal loading. **b.** Clonogenic survival of the NSCLC cell lines used in a) following exposure to the indicated doses of IR. Each point represents the mean of at least three independent experiments, each performed in triplicate ± SEM. **c.** ATM protein expression measured by western blot was quantified using the ImageJ software and plotted together with the surviving fraction at 2 Gy of the respective cell line. ATM protein expression values are normalised to β-Actin levels and expressed as the mean of control and IR-treated sample for each cell line. **d.** The increase in ATM autophosphorylation levels measured by western blot for each cell line was quantified and plotted as in c). ATM S1981 levels (60 min after 10 Gy IR) are normalised to β-Actin levels of the respective sample and expressed as a fold increase of the IR-treated sample compared with unirradiated control for each cell line.

As anticipated, the A-T fibroblast cell line GM05849 showed no ATM protein expression and no phosphorylation of the ATM downstream targets KAP1, SMC1, NBS1 or CHK2 in response to IR (Figure 2a). Notably, only low levels of total ATM protein were evident in the H1395 and H23 cell lines. All other cell lines showed levels of ATM protein expression that were comparable to the control cell lines COR-L105 or A549. Following exposure to IR, a robust increase in the levels of ATM activation and phosphorylation of several ATM downstream targets was evident in the A549, COR-L105 and H1299 control cell lines within 60 minutes post-irradiation. HCC-366, H1568, H2087, H1734 and H520 cells also displayed normal kinase activity for both ATM itself, as well as its downstream targets (Figure 2a).

Consistent with the low total ATM protein levels observed in H1395 and H23 cells, the radiation-induced kinase activity of ATM was severely impaired, as seen by an inability to induce ATM autophosphorylation or phosphorylation of the ATM downstream targets KAP1, SMC1 and NBS1 in response to IR. As the H23 cell line also harbours a mutation in the TP53 gene (p.M246I), aberrant levels of total and phosphorylated p53 were observed in this cell line (Figure 2a). No (H1299) or aberrant phospho- and total p53 protein levels were also observed in the other p53-mutated NSCLC cell lines (HCC-366, H1568, H1734, H2087 and H520) and the SV-40 transformed GM05849 cell line, independent of radiation treatment.

It should be noted, that the phosphorylation levels of several ATM downstream targets, like NBS1 or CHK2, differed between cell lines and not all downstream targets were phosphorylated to a comparable extent in each cell line. COR-L105, for example, displayed a marked increase in the phosphorylation of ATM, KAP1, SMC1 and CHK2, but only a modest increase in the phosphorylation of NBS1. H1568 cells on the other hand, showed only a modest increase in KAP1 phosphorylation levels, but a robust increase in pATM, pSMC1, pNBS1 and pCHK2 levels. These differences are likely due to differences in the cell biology that result from unique combinations of somatic mutations and epigenetic alterations causing cancer cell line specific levels of replication stress, genomic instability and activity levels of certain signalling pathways. Furthermore, several of the downstream targets of ATM are shared targets of other DDR kinases, such as DNA-PKcs and ATR. In order to assess the kinase activity of ATM following exposure to IR, it therefore seems plausible and necessary to determine the phosphorylation levels of a combination of several ATM downstream targets.

### ATM-deficient cancer cells are highly sensitive to IR

One of the most prominent phenotypes of ATM-deficient cells is a hypersensitivity to IR [7]. We therefore determined the survival and colony forming ability of the cell lines following exposure to IR. The clonogenic survival assays provided further evidence for a functional loss of ATM in H1395 and H23 cells, as both cell lines exhibited substantial radiosensitivity compared with the A549, COR-L105 or H1299 control cell lines. H2087, HCC-366, H520 and H1568 cells, on the other hand, showed similar IR-sensitivity to control cells or an intermediate sensitivity (Figure 2b). The H1734 cell line did not form colonies suitable for clonogenic survival assays and was thus excluded from this part of our study.

To assess whether there might be a correlation between radiosensitivity and the expression levels or activity of ATM, we quantified the protein expression data obtained from western blot analysis using the ImageJ software. ATM protein expression, or the -fold induction of ATM autophosphorylation after IR-exposure, were plotted against the surviving fraction of the cells at 2 Gy, calculated from clonogenic survival assays (Figure 2c, 2d). The data indicated a strong positive linear correlation between ATM protein expression levels and radiosensitivity (R = 0.8832). For the -fold induction of ATM autophosphorylation levels after IR-exposure and radiosensitivity, the correlation was less pronounced, yet still considerable (R = 0.6825). A similar correlation with radiosensitivity was found for KAP1 S824 levels 60 min after irradiation (R = 0.65). For pCHK2 Thr68 levels, however, no significant correlation was found (R = 0.31; Figure S2).

**Figure 3:**
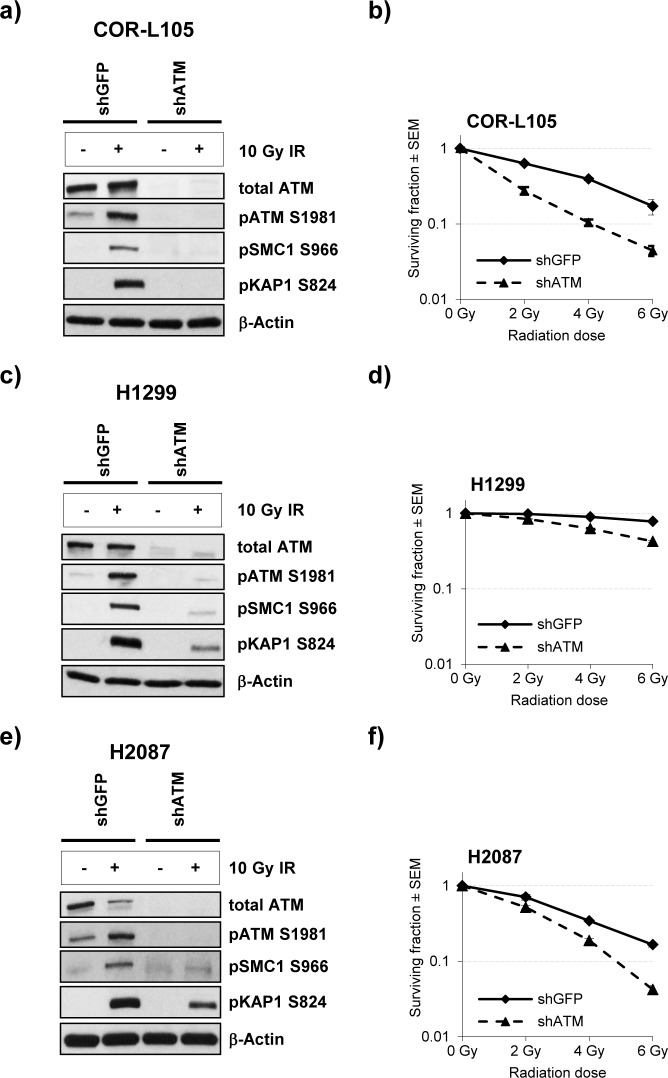
ATM knockdown in NSCLC cells reduces the IR-induced phosphorylation of ATM targets and increases radiosensitivity **a.**, **c.** and **e.** COR-L105, H1299 and H2087 cells, stably transfected with ATM short hairpin RNA (shATM) or the respective control plasmid (shGFP), were mock-irradiated (control) or irradiated with 10 Gy followed by preparation of protein lysates 60 min after irradiation. β-Actin was measured to ensure equal loading. **b.**, **d.** and **f.** Clonogenic survival of the indicated cell lines following exposure to IR. Each point represents the mean of three independent experiments, each performed in triplicate.

For further evaluation of the effect of ATM protein expression levels on radiation sensitivity, we compared the clonogenic survival of stable ATM knockdown cell lines and the corresponding controls following exposure of cells to IR (Figure 3). We found that ATM knockdown markedly increased the cytotoxic effects of IR in all cell lines, however, the effect was much greater in the cell line with the highest degree of ATM knockdown (COR-L105 - no detectable ATM protein or phosphorylation of ATM/KAP1/SMC1 following IR; Figure 3a, 3b) compared with those cell lines that showed low residual amounts of ATM protein and kinase activity (H2087 and H1299 - residual ATM, KAP1 and SMC1 phosphorylation; Figure 3c-3f).

Taken together these observations suggest that expression levels of ATM protein correlate well with the sensitivity of cancer cell lines to IR, and might thus be a useful biomarker to predict responses to radiotherapy. Assessment of the phosphorylation of ATM downstream targets, on the other hand, appears to be less reliable in the prediction of radiosensitivity, which could be due to differences in phosphorylation kinetics between cell lines or overlap with other DDR pathways, which converge in the same downstream targets (e.g. DNA-PKcs or ATR signalling).

In order to validate that the observed high degree of radiosensitivity in H1395 and H23 cells was due to impaired ATM function, we assessed clonogenic survival of cells treated with a combination of IR and the specific ATM inhibitor KU55933, or the DNA-PKcs inhibitor NU7441, which served as a radiosensitizer control [30]. We hypothesised that, if the observed radiosensitivity of H1395 and H23 cells was due to defects in ATM signalling, treatment of cells with an ATM inhibitor would produce no additional effect on radiation-induced cytotoxicity in these cells.

**Figure 4:**
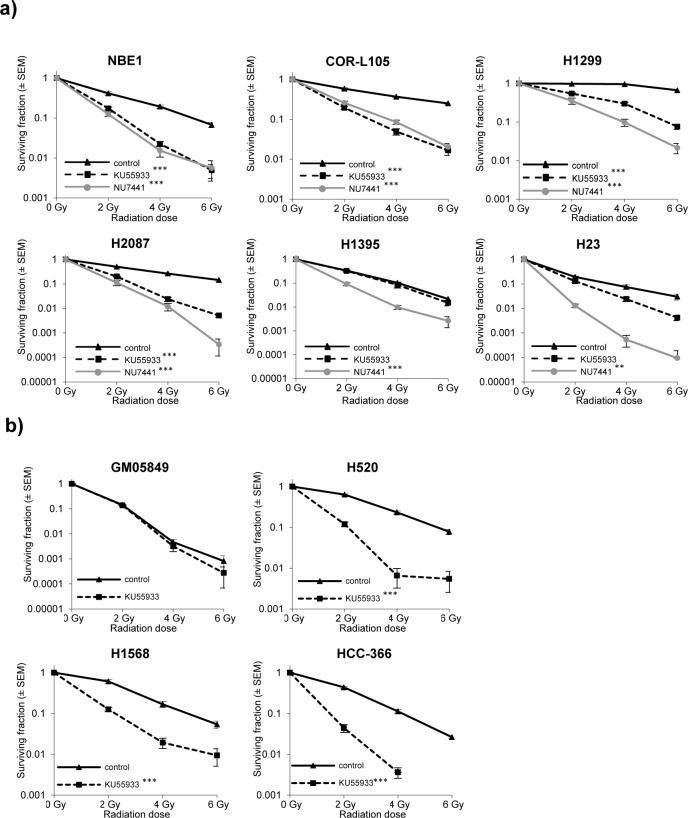
ATM-deficiency confers sensitivity to IR, which is not further increased by addition of the ATM inhibitor KU55933 **a.** Clonogenic survival of normal bronchial epithelial (NBE1) and NSCLC cells following exposure to IR alone, or in combination with ATM (KU55933; 10 μM) or DNA-PK (NU7441; 500 nM) inhibition. Cells were pre-treated with the inhibitors for 1 h prior to irradiation and treatment was continued for 24 h. Each point represents the mean of three independent experiments, each performed in triplicate. Statistically significant differences (univariate ANOVA) between survival curves are indicated with ** = *p*-values < 0.001 and *** = *p*-values < 0.0001. **b.** Clonogenic survival of GM05849, HCC-366, H520 and H1568 cells following exposure to IR alone, or in combination with the ATM inhibitor KU55933 (10 μM). Treatment, display of results and statistical analysis as in a).

Consistent with loss of ATM function, exposure of H1395 and H23 cells to the ATM inhibitor did not significantly further increase the cytotoxic effects of IR (H23 *p* = 0.06; H1395 *p* = 0.8) (Figure 4a). The small degree of radiosensitisation by KU55933 observed in H23 cells (*p* = 0.06) is possibly due to residual amounts of functional ATM in this cell line. Normal bronchial epithelial cells (NBE1), as well as COR-L105, H1299 and H2087 cells, on the other hand, were profoundly radiosensitised by KU55933 treatment (*p*-values < 0.001 for all four cell lines). DNA-PKcs inhibition significantly radiosensitised all cell lines (Figure 4a; *p*-values < 0.001 for all cell lines). We also determined the effect of pharmacological ATM inhibition on the radiosensitivity of the remaining ATM-mutated cell lines. In all three cell lines (HCC-366, H520, H1568), KU55933 treatment significantly increased the radiation-induced cytotoxicity (*p*-values < 0.001 for all three cell lines), while the ATM-negative GM05849 fibroblast cell line showed no radiosensitisation (*p* = 0.993; Figure 4b).

### ATM-deficient cancer cells exhibit cell cycle checkpoint defects

Loss of ATM function is known to lead to defects in cell cycle checkpoint activation in response to DNA damage. In particular, these checkpoint defects include an impaired cell cycle arrest at the G1/S boundary following DNA DSB induction, which is also a characteristic of p53-deficient cells [31], as well as an inability to slow on-going DNA replication in cells exposed to IR, a phenomenon described as radiation-resistant DNA synthesis [32–34].

**Figure 5:**
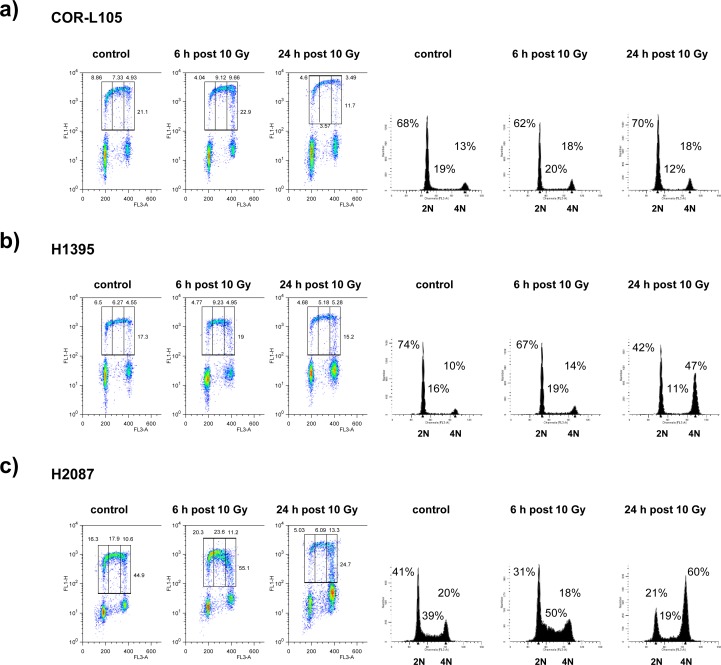
ATM deficiency leads to attenuation of the G1/S and intra-S-phase checkpoints Asynchronous populations of **a.** COR-L105, **b.** H1395 and **c.** H2087 cells were mock-irradiated or irradiated with 10 Gy. After irradiation, cells were incubated at 37°C for the indicated times before being pulse-labelled with BrdU for 20 min. Cells were then harvested, fixed, and stained with PI and anti-BrdU antibody for FACS analysis. BrdU staining (left-hand panels) and PI-staining (right-hand panels) are representative of two and at least three independent experiments, respectively. The upper gates represent (from left to right) early-S-, intra-S- and late-S- /early G2-phase cell populations.

To further evaluate potential defects in ATM function, we analysed cell cycle checkpoint activation in cells exposed to IR, using flow cytometry. We performed BrdU pulse labelling of COR-L105, H1395 and H2087 cells to detect S-phase populations following IR treatment (Figure 5). In COR-L105, a reduction in the proportion of BrdU-labelled cells in early S-phase within 6 h post-irradiation was evident, indicating activation of the G1/S checkpoint, which prevents cells with damaged DNA from entering S-phase and is typical for p53 and ATM wt cells (Figure 5a). In contrast, H1395 and H2087 cells showed no or only a minimal decrease in BrdU-labelled cells in early S-phase after irradiation, suggesting that these cells failed to induce the G1/S cell cycle checkpoint, consistent with a functional impairment of ATM or p53. Over time, an accumulation of cells in G2 was evident in both cell lines (Figure 5b, 5c), representing the activation of a G2/M cell cycle arrest following IR. While H2087 cells displayed a significant increase in the overall S-phase population during early time-points after IR, indicating a slowdown of S-phase progression, this effect was less pronounced in H1395 cells (Figure 5b), consistent with a defect in ATM signalling in H1395, resulting in radioresistant DNA synthesis.

Taken together these results demonstrate that the homozygous p.T2666A (H1395) and p.Q1919P (H23) missense substitutions in the ATM gene lead to a strong reduction in ATM protein levels and severe impairment of the ATM signalling pathway, indicating that these are deleterious mutations. The NSCLC cell line H2087 has been reported to carry two heterozygous ATM missense substitutions (p.E848Q and p.L2965F) [22–24]. According to PolyPhen-2 one of these mutations was predicted to be benign, while the other one was likely to be damaging (Table 1). However, we were not able to verify the presence of the potentially damaging p.L2965F mutation and our results demonstrate that this cell line exhibits normal ATM-dependent signalling in response to IR, as well as increased radiosensitivity following treatment with an ATM inhibitor or knockdown of ATM protein. These observations suggest that the ATM missense substitution present in this cell line is either a benign passenger mutation, or, if it is a deleterious mutation, that the remaining wildtype allele allows for sufficient expression of functional ATM to retain activity. Similar results were obtained in four other NSCLC cell lines, which have been reported to harbour ATM missense changes, some of which were predicted to be damaging according to PolyPhen-2 (HCC-366, H1568, H1734 and H520; Table 1). Altogether, the PolyPhen-2 prediction only supported the results from phenotypic analyses in the case of 4 out of 7 ATM mutations (57%). However, as the reported ATM mutations in these cell lines are heterozygous (with allele frequencies between 0.23 and 0.78) [23], it is possible that the remaining wildtype ATM alleles are sufficient to allow for normal ATM expression levels and function. Supportive evidence for this hypothesis comes from studies in patients with chronic lymphocytic leukaemia containing a deletion of the ATM gene locus (11q deletion) which demonstrated, that the remaining ATM wildtype allele had to be mutated in order to see a functional impairment of ATM signalling [35]. This raises the question, whether heterozygosity of ATM mutations in cancer cells is generally associated with retained ATM activity. Future studies will have to address this question and establish, whether mutant ATM protein can act in a dominant-negative way and if so, which mutations would lead to this phenotype. Nevertheless, our results suggest that in the case of heterozygous ATM mutations, software tools like PolyPhen-2 are only of limited use to predict the phenotypic consequences of a given missense mutation, due to the recessive nature of functional loss in the case of ATM. Our results thus demonstrate, that the occurrence of a somatic ATM mutation in a cancer cell does not imply an impairment or loss of ATM function.

When performing functional studies, we focussed on the activity and functionality of the ATM signalling pathway in response to IR, as radiotherapy is one of the most commonly used therapies for lung cancer patients and the ATM-dependent response of cells to IR has been well characterised. However, it is possible that some of the mutations only affect the binding of ATM to certain substrates, which would consequently only alter certain arms of the ATM signalling network or the activity of ATM in a specific context, for example in response to reactive oxygen species [26, 36]. As ATM has been reported to have approximately 1000 downstream targets [37], extensive phospho-proteomic studies would be required to investigate the effect of each ATM mutation on the overall activity of ATM.

### Identification of ATM-deficiency in a clinical setting

As ATM-deficiency confers a high sensitivity to IR, radiomimetic drugs and topoisomerase inhibitors [7, 16, 17], it would be of clinical significance - and help guide the process of identifying the best treatment option for patients - to be able to differentiate between benign and deleterious, as well as homo- and heterozygous, mutations. However, the ATM gene extends over approximately 150 kb of genomic DNA, contains 66 exons and encodes for a 3056 amino acid protein [38]. This large size of the ATM gene renders routine DNA sequencing a challenging diagnostic tool to identify patients with ATM-deficiency and stromal contamination of samples hinders the determination of zygosity. Furthermore, as our results clearly show, the presence of an ATM mutation does not necessarily imply that there will be a functional impairment of ATM signalling, particularly in the case of heterozygous missense changes. Therefore, we aimed to address the question, how patients with tumoral loss of ATM function could be identified in a clinical setting.

Studies on ATM missense mutations found in A-T patients have shown that the majority of missense mutations which lead to functional impairment of ATM do so by de-stabilising the protein, leading to reduced or absent protein levels [18, 27, 28, 39–41]. Our data suggests that the same principle may apply to cancer-associated ATM missense mutations, as we identified two NSCLC cell lines harbouring homozygous missense mutations in the ATM gene (H1395 and H23) with impaired ATM function. In both cases, mutation was associated with markedly reduced ATM protein expression. To our knowledge, mutations leading to expression of a kinase- dead (kd) ATM protein have only rarely been reported in cancer [42] and none of them in NSCLC.

**Figure 6:**
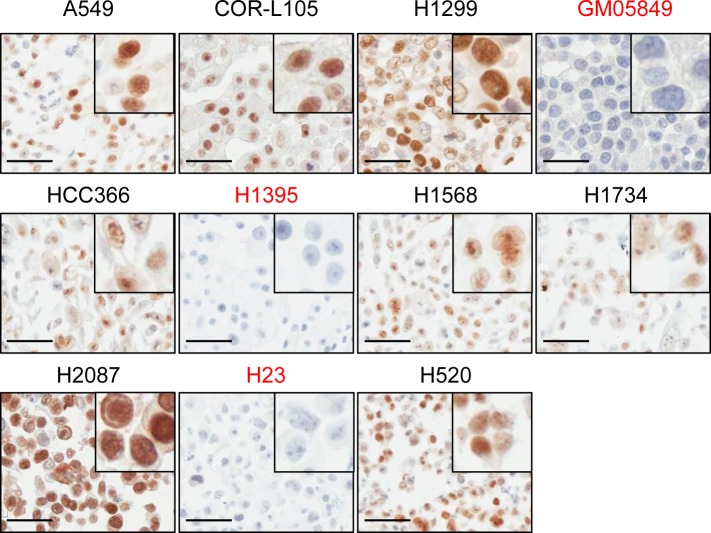
ATM protein expression in NSCLC cell lines assessed by immunohistochemistry A panel of 35 NSCLC cell lines was analysed for ATM protein expression by IHC. The A-T fibroblast cell line GM05849 was included as ATM-negative control. Representative images of the cell lines used in this study are shown. Pictures were taken at a 400x magnification. Scale bar = 50 μm. A detail magnification is shown in the upper right corner of each picture.

To address this possibility further, we evaluated immunohistochemical (IHC) staining of ATM in human tumour samples as a potential method for the identification of patients with a loss or reduction of ATM function. For the optimisation of the staining procedure we generated a cell line microarray from paraffin-embedded cell pellets derived from a panel of 35 NSCLC cell lines. The A-T fibroblast cell line GM05849 was included as ATM-negative control. Following IHC labelling of ATM, we detected specific nuclear staining in 33/35 NSCLC cell lines, including HCC-366, H1568, H1734, H2087 and H520 cells. No staining for ATM could be observed in GM05849 cells and in 2/35 NSCLC cell lines (5.7%) - H23 and H1395 (Figure 6). This is in agreement with our findings from western blot analyses, which demonstrated a loss of ATM protein expression in both cases, and confirms the specificity of the antibody on paraffin-embedded material.

### ATM protein expression is frequently lost or reduced in human NSCLC and colorectal cancer

**Figure 7:**
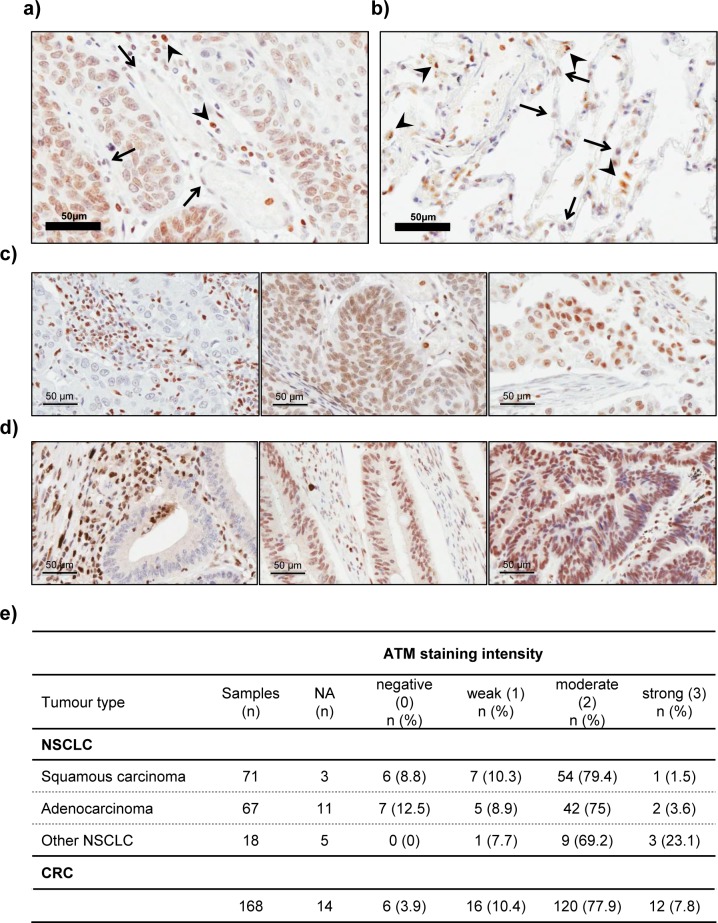
Immunohistochemical analysis of ATM protein expression in lung tissue, NSCLC and CRC samples **a.** Representative image of a lung adenocarcinoma specimen with moderate ATM protein expression, in which the nuclear staining for ATM in tumour cells is similar to that observed in stromal cells (arrows). Lymphocytes (arrowheads) were used as basis for strong staining intensity. **b.** Representative image of ATM IHC staining in lung tissue. Note the higher staining intensity in resident macrophages (arrowheads), compared to pneumocytes (arrows). **c.** and **d.** Representative images of negative (left), moderate (middle) and strong (right) staining for ATM in NSCLC **c.** and colorectal cancer **d.** specimens. Pictures were taken at a 400x magnification. Scale bar = 50 μm. **e.** Table summarising the results of ATM protein expression analysis in 156 human NSCLC and 168 CRC samples by immunohistochemistry. NA = excluded from analysis due to poor staining or tissue quality (*n* = 19 for NSCLC and *n* = 14 for CRC).

We then proceeded with the analysis of ATM protein expression in human NSCLC tumour specimens (*n* = 156) by IHC. The staining intensity of tumour cells was graded relative to the intensity observed in the tumour stroma (e.g. endothelial cells or fibroblasts), which was used as the reference to categorise moderate staining in each case (Figure 7a). The intensity of staining in tumour cells was scored independently by two observers on a scale of 0-3 (0 = negative, 1 = weaker than stroma, 2 = same as stroma or moderate, and 3 = stronger staining than in stroma). Specimens, in which no or only very weak staining was observed in the tumour stroma were excluded from analysis (NA; *n* = 19/156). Tumour adjacent non-cancerous lung tissue was stained to assess ATM protein expression in the lung parenchyma under physiological conditions (*n* = 8; Figure 7b). Lymphocytes and macrophages show higher ATM staining than stromal or lung parenchyma cells (e.g. pneumocytes) and were therefore, where present, considered a measure for high staining intensity (Figure 7b).

The scoring results are summarised in Figure 7e. A loss of ATM protein expression in tumour cells was evident in 13/137 (9.5%) cases, (Figure 7c, 7d), a proportion which is at the top of the range of reported ATM mutations in NSCLC (5.1-9.1%; [14, 15]). This could be due to the small sample size in our study. Moreover, in addition to point mutations, loss or rearrangement of the ATM gene locus or epigenetic silencing of gene expression could also contribute to the loss of ATM protein expression. Indeed, hypermethylation of the ATM promoter resulting in decreased protein levels and increased radiosensitivity has been described for colorectal and glioma cell lines [43, 44]. Furthermore, studies in locally advanced breast cancer have shown that the ATM gene is a target for epigenetic silencing [45].

We further evaluated the clinical utility of this method for identifying cancer patients with tumoral loss of ATM function, by analysing ATM protein expression in 168 human colorectal cancer specimen. ATM immunohistochemistry was performed on tissue microarrays containing quadruple cores of each sample. 14 of the 168 samples were excluded from analysis due to poor stromal staining or insufficient tumour material. Scoring of ATM staining intensity was performed as for the lung cancer specimens. Conversely to our data obtained in NSCLC, the proportion of tumours exhibiting no detectable ATM protein expression (6/154; 3.9%) in our study was below the reported mutation rate for ATM in this cancer type (approx. 11.3%) [10], raising the possibility that heterozygous mutations or passenger mutations in ATM might be common in CRC. This finding also highlights the importance of performing additional assays (e.g. IHC for ATM protein expression) and functional studies to support sequencing data. Notably, several of the cancer types with the highest reported frequency of ATM mutations (including bladder cancer, melanoma, CRC and NSCLC) also show the highest overall rate of somatic mutations and are therefore likely to have a higher load of passenger mutations [46].

We conclude that immunohistochemical analysis of ATM protein expression in tumour samples, for example in tumour biopsies, may be a valuable tool to identify cancer patients with tumoral loss of ATM function. It would not identify the rare cases where a mutation causes expression of a kinase dead protein [42], but it would allow for the detection of patients with loss of ATM function due to deletion of the gene locus, epigenetic changes or mutations that lead to loss of ATM expression, including truncating, missense or frameshift mutations, while excluding patients with either heterozygous or benign ATM mutations. As hypersensitivity to ionising radiation is one of the most prominent phenotypes of ATM-deficient cells [7] and given the observed increased sensitivity of A-T cells to topoisomerase inhibitors (e.g. etoposide or irinotecan) [16, 17], this may help to identify patients that could get increased benefit from these therapies. In addition to a potential role in the response of tumours to chemo- or radiotherapy, ATM-deficiency may also identify a patient subset that could benefit from small molecule inhibitors in the context of inducing a synthetic lethal response, an approach for personalised cancer medicine which has gained much popularity in recent years [47]. IHC analysis of ATM expression could thus help guide the process of deciding on optimal cancer treatment, especially in those cases where radiotherapy is an option.

## MATERIALS AND METHODS

### Cell culture

Telomerase immortalised normal bronchial epithelial cells (LIMM-NBE1) were kindly provided by Dr. Pamela Rabbitts (Leeds Institute of Molecular Medicine, University of Leeds, UK) [48]. GM05849 A-T fibroblasts and COR-L105 cells were obtained from the Coriell Institute and the European collection of cell cultures, respectively. All other cell lines were obtained from ATCC. Cells were maintained in advanced DMEM/F12 (Life technologies), supplemented with 5% fetal bovine serum (Sigma), 2 mM GlutaMAX^TM^ and 50 U penicillin/50 μg/ml streptomycin (Life technologies) in a humidified incubator at 37°C and 7.5% CO_2_. Cell line identification was carried out by sequencing of highly polymorphic loci in the mitochondrial DNA as described in [49]. Mycoplasma testing was performed using a real-time PCR-based assay [50].

### Stable knockdown of ATM

The shRNA target sequence for ATM 5′- GCAACATACTACTCAAAGA -3′ and the control sequence (shGFP) 5′- GCATGGATGAACTATACAA -3′ were cloned into the pSuperRetro.puro vector (Oligoengine). Transfections were carried out with Lipofectamine 2000 (Life technologies) as described by the manufacturer. Following a 14-day selection period with puromycin (Sigma), single colonies were picked, expanded and tested for gene knockdown using western blot analysis. Stably transfected cells were maintained in culture medium supplemented with 0.5 μg/ml puromycin. At least 24 h prior to any experiments, the selection medium was removed and replaced by puromycin-free culture medium.

### ATM sequencing

The regions of interest from the ATM gene were pre-amplified from genomic DNA as previously described [40]. The PCR amplification was performed in 25 μl reactions containing 2 units MiFi DNA polymerase (Bioline Reagents Ltd), 0.1 μg genomic DNA and 0.5 μM of each primer in 1x MiFi reaction buffer. Sanger DNA sequencing of the resulting PCR-products was performed commercially (SourceBioscience). For a computational assessment of the possible impact of the identified amino acid substitutions on ATM structure and function, the PolyPhen-2 software tool was used [25], accessed through the web interface at http://genetics.bwh.harvard.edu/pph2/index.shtml.

### Drug treatments and irradiation

NU7441 and KU55933 (Selleck chemicals) were prepared in DMSO (Sigma) at a concentration of 10 mM and stored at −20°C. Irradiations were carried out using a GSR D1 irradiator containing a ^137^Cs source (Gamma-Service Medical GmbH).

### Western blotting

Cell lysates were prepared on ice in 50 mM Tris-HCl pH 7.6, 137 mM NaCl, 10% glycerol, 0.1% Igepal, 0.1% SDS, 50 mM NaF, and 1 mM sodium orthovanadate (Sigma), supplemented with cOmplete protease inhibitors (Roche), and homogenised by brief sonication. 50 μg of protein were resolved by SDS-PAGE and transferred to nitrocellulose membranes. Antibodies used were CHK2 Thr68 and p53 S15 (#2661, #9284; Cell Signaling Technologies), KAP1 S824, and SMC1 S966 (#A300-274A, #A300-767A, #A300-050A; Bethyl Laboratories), p53 (#sc-126; Santa Cruz Biotechnology), NBS1 S343 (#ab47272; Abcam), ATM S1981 (#2152-1; Epitomics Inc.), ATM (#04-200; Upstate Millipore), α-Tubulin and β-Actin (#T6074, #A1978; Sigma). After incubation with primary antibody overnight at 4°C, membranes were probed with HRP-conjugated secondary antibodies. Detection was carried out by chemiluminescence and scanning on a ChemiDoc^TM^ chemiluminescence imager (BioRad). Densitometric quantification of protein bands was performed using the ImageJ software.

### Clonogenic survival assay

Cells were plated in triplicate into 6-well plates and allowed to re-attach (depending on the cell line for 8 to 16 h). 1 h prior to irradiation, cells were pre-incubated with vehicle (DMSO), NU7441 (500 nM) or KU55933 (10 μM). 24 h post-irradiation, cells were washed with PBS and then incubated in drug-free culture medium to allow for colony formation. When colonies reached a size of ≥50 cells (7 to 21 days depending on the cell line), they were fixed and stained with crystal violet/methanol, air-dried and counted. Data were calculated as fraction of surviving colonies relative to mock-irradiated vehicle control or inhibitor treated cells (± SEM).

### Cell cycle analysis

Cells were harvested by trypsinisation and fixed in 70% ethanol (4°C). Cells were washed in PBS and incubated in PBS containing 100 μg/ml RNaseA (Qiagen) for 30 min at 37°C. Propidium iodide (PI, Calbiochem) was added at a final concentration of 20 μg/ml. DNA content was determined using a FACScan Flow Cytometer (BD Biosciences) and BD CellQuestTM Pro Software. The proportion of cells in G1, S or G2/M was determined using ModFit LT software (Verity Software House).

### BrdU incorporation analysis

Asynchronous cell populations were mock-irradiated or irradiated (10 Gy). 6 h or 24 h after irradiation, cells were pulse-labelled with 20 μM BrdU (Sigma) for 20min. After harvesting and fixation in 70% ethanol (4°C), cells were re-suspended in 2 M HCl and incubated for 20 min at RT. Cells were first washed in PBS, then PBS containing 2% fetal bovine serum (FBS; Sigma), before re-suspension in PBS+2% FBS containing mouse anti-BrdU antibody (#347580; BD Biosciences). Cells were incubated in primary antibody for 90 min at RT, washed in PBS+2% FBS and then incubated in goat anti-mouse Alexa 488 secondary antibody (Invitrogen) for 1 h at RT, before re-suspension in PBS containing PI. DNA content and BrdU incorporation were measured using a FACSCalibur Flow Cytometer (BD Biosciences) and BD CellQuestTM Pro Software. Analysis of the data was carried out using the FlowJo software (Tree Star Inc.).

### Immunohistochemistry (IHC)

IHC analysis of ATM protein expression was carried out on 5 μm sections of paraffin-embedded NSCLC specimens (ProteoGenex, Inc., and the Oxford Radcliffe Biobank), a lung carcinoma and normal tissue array (#BC041115a; US Biomax, Inc.) and tissue microarrays containing quadruple cores from 168 colorectal cancer specimen derived from the COIN clinical trial [51]. Antigen retrieval was performed in 10 mM Tris, 1 mM EDTA, 0.05% Tween20; pH 9. The EnVision+ detection system (#K4065; Dako) was used for staining according to the manufacturer's instructions with the addition of a blocking step in protein blocking solution (#ab64226; Abcam) performed for 1 h at room temperature prior to the incubation of tissues in antibody solutions. Primary antibody specific for ATM (clone Y170; #04-200; Millipore) was incubated with the sections overnight at 4°C. Stained slides were scanned (Aperio ScanScope CS) utilising the ScanScope software (Aperio). The staining intensity of tumour cells was scored relative to the intensity observed in the tumour stroma, which served as intrinsic positive control for each sample and was used as reference for moderate staining in each case. The intensity of staining was graded as negative, weak, moderate or strong.

### Data analysis and statistics

All values are expressed as the mean ± SEM of at least three independent experiments unless otherwise stated. Drug effects on radiosensitivity were compared by univariate analysis of variance (ANOVA; SPSS 22.0) to detect statistically significant differences between drug+IR and IR-only treated controls. Differences were considered significant for values of *p* < 0.01.

## SUPPLEMENTARY MATERIALS FIGURES


