# Supplementary material for: Phenotypic consequences of somatic mutations in the ataxia-telangiectasia mutated gene in non-small cell lung cancer
Source: Oncotarget. 2016 Sep 2;7(38):60807–22. doi: 10.18632/oncotarget.11845 (PMC5308618; doi:10.18632/oncotarget.11845)
Supplement: Supplementary file 1 [file oncotarget-07-60807-s001.pdf]

Phenotypic consequences of somatic mutations in the ataxia-telangiectasia mutated gene in non-small cell lung cancer

Supplementary Material

Supplementary Figure S1

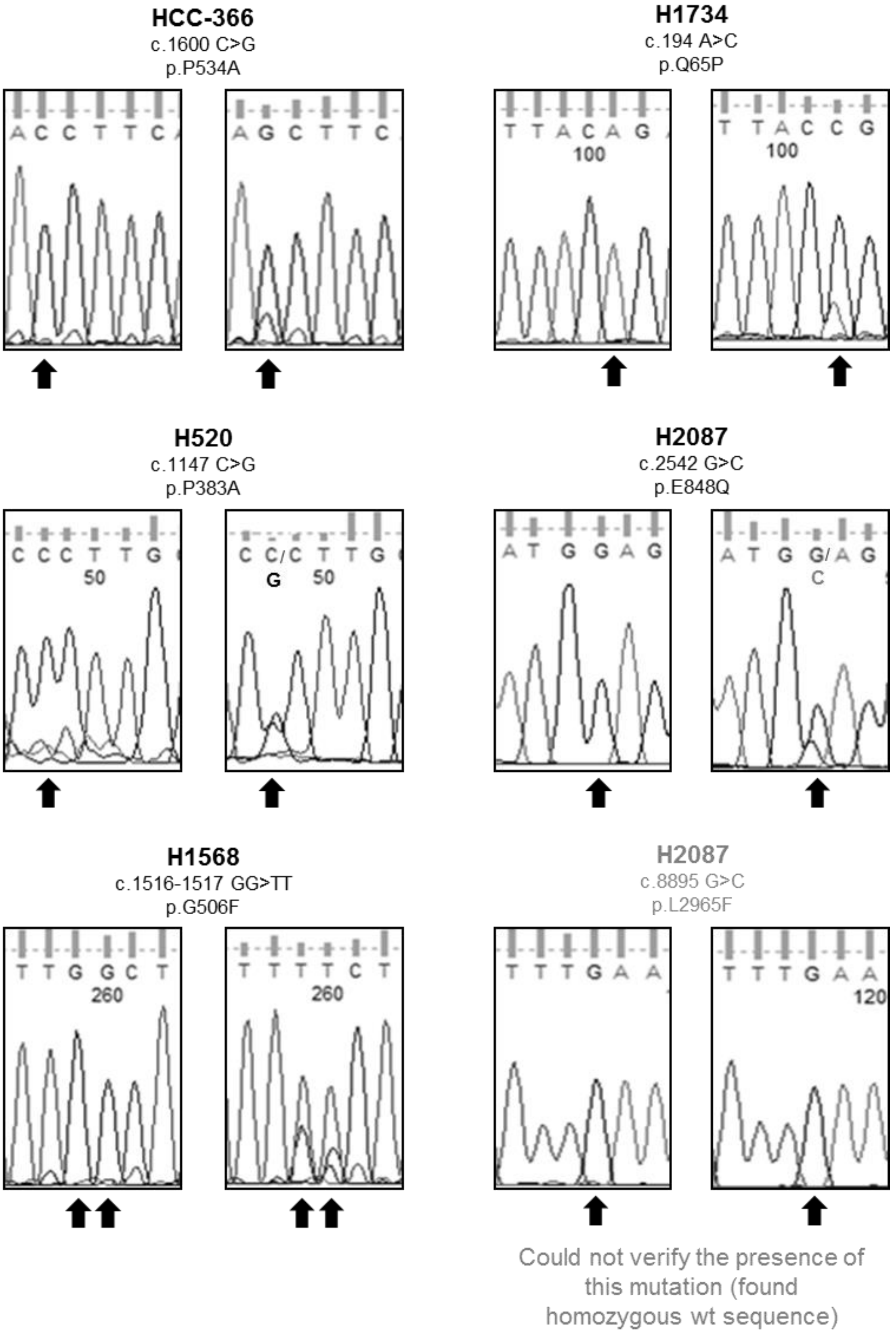

Supplementary Figure S1: DNA sequencing results showing the presence of the reported missense mutations in the ATM gene in the NSCLC cell lines used in this study. Wildtype sequences derived from H1299 or COR-L105 cells are shown on the left. Arrows indicate the positions of the respective missense change.

Supplementary Figure S2

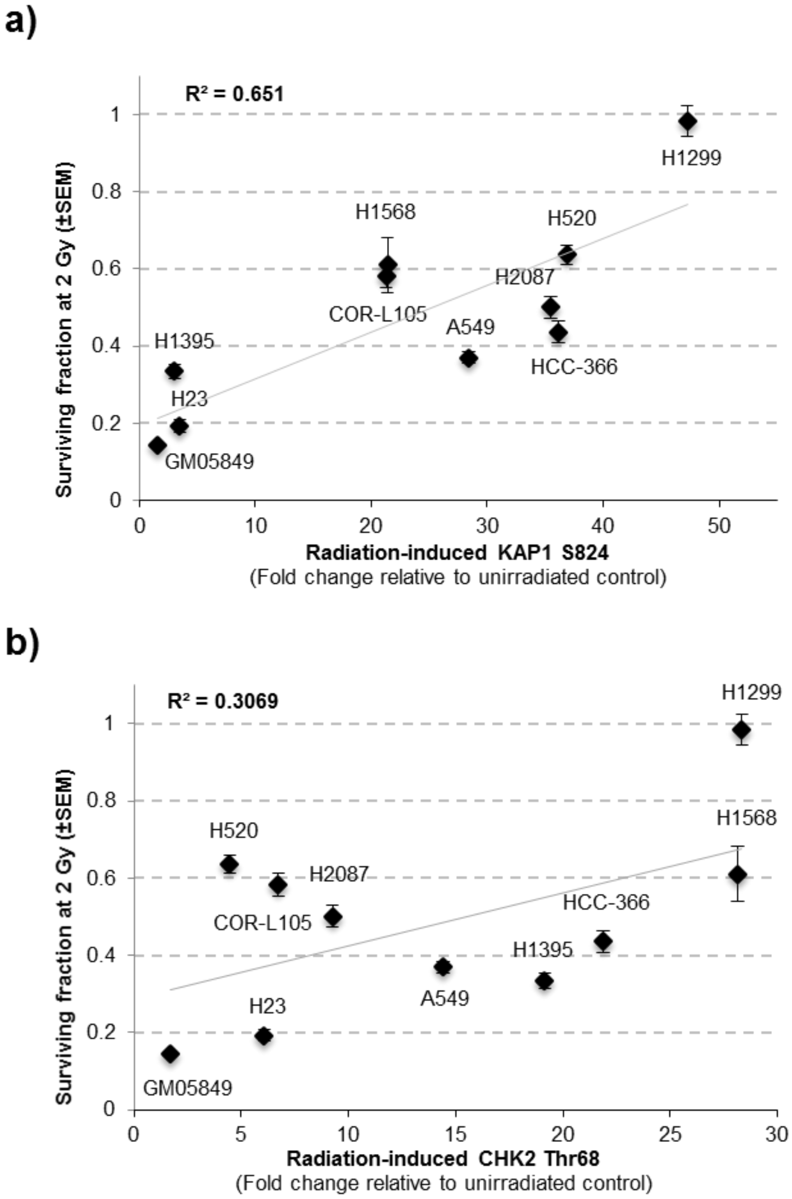

Supplementary Figure S2: The -fold increase in **a)** KAP1 (S824) and **b)** CHK2 (Thr68) phosphorylation levels 60 min after exposure of cells to 10 Gy IR - as

measured by western blot - were quantified using the ImageJ software. Levels of the respective phospho-protein are normalised to β-Actin levels of the respective sample and expressed as a -fold increase of the IR-treated sample compared with unirradiated control for each cell line. Values are plotted together with the surviving fraction at 2 Gy of the respective cell line. The R^2 values were calculated to assess a possible correlation between –fold increase in phosphorylation levels of the respective protein and sensitivity of cells to IR.
